# Evaluation of multicomponent recombinant vaccines against *Actinobacillus pleuropneumoniae *in mice

**DOI:** 10.1186/1751-0147-52-52

**Published:** 2010-09-11

**Authors:** Meili Shao, Yong Wang, Chunlai Wang, Yang Guo, Yonggang Peng, Jiandong Liu, Guangxing Li, Huifang Liu, Siguo Liu

**Affiliations:** 1 Division of Bacterial Diseases, National Key Laboratory of Veterinary Biotechnology, Harbin Veterinary Research Institute, Chinese Academy of Agricultural Sciences, Harbin 150001, China; 2 Northeast Agricultural University, Harbin 150030, China; 3 Shenyang Agricultural University, Shenyang 110161, China

## Abstract

**Background:**

Porcine contagious pleuropneumonia (PCP) is a highly contagious disease that is caused by *Actinobacillus pleuropneumoniae *(APP) and characterized by severe fibrinous necrotizing hemorrhagic pleuropneumonia, which is a severe threat to the swine industry. In addition to APP RTX-toxins I (ApxI), APP RTX-toxin II (ApxII), APP RTX-toxin III (ApxIII) and Outer membrane protein (OMP), there may be other useful antigens that can contribute to protection. In the development of an efficacious vaccine against APP, the immunogenicities of multicomponent recombinant subunit vaccines were evaluated.

**Methods:**

Six major virulent factor genes of APP, i.e., *apxI*, *apxII*, *apxIII*, APP RTX-toxins IV (*apxIV*), *omp *and type 4 fimbrial structural (*apfa*) were expressed. BALB/c mice were immunized with recombinant ApxI ( rApxI), recombinant ApxII (rApxII), recombinant ApxIII (rApxIII) and recombinant OMP (rOMP) (Group I); rApxI, rApxII, rApxIII, recombinant ApxIV (rApxIV), recombinant Apfa (rApfa) and rOMP (Group II); APP serotype 1 (APP1) inactivated vaccine (Group III); or phosphate-buffered saline (PBS) (Control group), respectively. After the first immunization, mice were subjected to two booster immunizations at 2-week intervals, followed by challenge with APP1 Shope 4074 and APP2 S1536.

**Results:**

The efficacy of the multicomponent recombinant subunit vaccines was evaluated on the basis of antibody titers, survival rates, lung lesions and indirect immunofluorescence (IIF) detection of APP. The antibody level of Group I was significantly higher than those of the other three groups (*P *< 0.05). The survival rate of Group I was higher than that of Groups II and III (*P *< 0.05) and the control (*P *< 0.01). Compared with the other three groups, the lungs of Group I did not exhibit obvious hemorrhage or necrosis, and only showed weak and scattered fluorescent dots by IIF detection.

**Conclusion:**

The result indicates that the multicomponent recombinant subunit vaccine composed of rApxI, rApxII, rApxIII and rOMP can provide effective cross-protection against homologous and heterologous APP challenge.

## Background

Porcine contagious pleuropneumonia (PCP) is a highly contagious disease that is caused by *Actinobacillus pleuropneumoniae *(APP) and characterized by severe fibrinous necrotizing hemorrhagic pleuropneumonia [1], which is a severe threat to the swine industry.

At present, an inactivated whole cell vaccine derived from APP is used for PCP prevention in many countries [2,3]. However, the protection provided by the inactivated vaccine is not sufficient [4,5], for the reason that the inactivated vaccine rarely contains exotoxins excreted to the medium by the bacteria during growth [6-8]. In addition, some protein components may be damaged or lost during the inactivation process. Several studies have shown that effective protection can be provided by combined subunit vaccines composed of virulence factors of APP [9,10], such as transferrin-binding protein, lipoprotein [11], capsular polysaccharide [CPS] or lipopolysaccharide [LPS] [12]. Combined subunit vaccines, such as the multicomponent vaccine composed of APP RTX-toxins I (ApxI), APP RTX-toxin II (ApxII), APP RTX-toxin III (ApxIII) and Outer membrane protein (OMP), can provide higher protective efficacy against challenge with 12 serotypes of APP [13,14], which demonstrates that the development of multicomponent subunit vaccines should be pursued further.

In addition to ApxI, ApxII, ApxIII and OMP, there may be other useful antigens that can contribute to protection. As an important virulent factor, the pilus has excellent immunogenicity among many Gram-negative bacteria [15-17]. The enterotoxigenic CS4 pilus of *Escherichia coli *(*E. coli*) [18] and the toxin-coregulated pilus (TCP) of *Vibrio cholerae *[19] have been chosen as candidate antigens for subunit vaccines. The type 4 fimbrial structural gene (*apfA*) of APP was shown to be present and highly preserved in different serotypes of APP [20,21], which suggests that the pilus of APP may have potential to be a component for vaccine preparation.

APP RTX-toxin IV (ApxIV) toxin is another potentially valuable antigen that has been identified within recent years as an APP toxin. The ApxIV toxin was shown to be the only toxin that can be produced by all serotypes of APP and is only expressed in vivo during infection. Moreover, ApxIV toxin can stimulate a high level of antibody [22]. These findings indicate that ApxIV toxin may be responsible for cross-protection in pigs that have recovered from natural infection and are resistant to reinfection with any other serotype of APP.

In this study, we cloned and expressed ApxI, ApxII, ApxIII toxins, OMP as well as the Apfa and ApxIV toxin of APP. On the basis of these recombinant antigens, different multi-component recombinant vaccines were made, and the efficacy of these vaccines was evaluated in order to determine whether the Apfa toxin can contribute to the protective immunity of a recombinant subunit vaccine.

## Materials and methods

### Bacterial strains, growth conditions, vectors and sera

The APP serotype 1 reference strain Shope 4074, APP serotype 2 reference strain S1536 and *E. coli *BL21 were obtained from the Chinese Institute of Veterinary Drug Control (IVDC); the prokaryotic expression vector pGEX-6P-1 was purchased from Invitrogen (Carlsbad, CA, USA). Rabbit antisera were produced by immunization of rabbits with inactivated APP1 and APP2; the immunization was performed by multipoint subcutaneous injections, and the immunization schedule comprised three immunizations at 2-week intervals. Ten days after the third immunization, blood was collected and the serum was separated and stored in our laboratory. The APP was grown in beef heart infusion broth or agar supplemented with 10% horse serum and 100 μg/ml Nicotinamide Adenine Dinucleotide (NAD), and the *E. coli *BL21 strain was grown in Luria-Bertani (LB) broth or agar containing 50 μg/ml ampicillin.

### Mice

Male BALB/c mice (*n *= 80), aged 6 weeks, were purchased from Harbin Medical University. Animal experiments were performed in accordance with the guidelines of Chinese Council on Animal Care. The research protocol was approved by Harbin Veterinary Research Institute Committees on Biosafety.

### Expression and purification of recombinant proteins

The genes *apxIA*, *apxIVA*, *apfa *and *omp *were amplified from the APP1 Shope 4074 genome; *apxIIA *and *apxIIIA *were amplified from the APP2 S1536 genome by PCR according to the reaction conditions shown in Table 1.

**Table 1:** Primers, sequences and PCR conditions used for the amplification of *apxIA*, *apxIIA*, *apxIIIA*, *apxIVA, omp and apfa *from *Actinobacillus pleuropneumoniae*

Genes	Primer sequences (5'-3')	Annealing temperature	Size of PCR product (bp)
*apxIA*	Forward: GCGGGATCCAACTCTCAGCTCGATAG	55°C	2520
	Reverse: GATGC**GTCGAC**AGCAGATTGTGTTAAAT		
*apxIIA*	Forward: GCGGGATCCATGTCAAAAATCACTT	54°C	2721
	Reverse: GC*GAATTC*AGCGGCTCTAGCTAAT		
*apxIIIA*	Forward: ACGGGATCCTGGTCAAGCATGTTAG	52°C	3114
	Reverse: ATGC**GTCGAC**TGCTCTAGCTAGGTTACC		
*apxIVA*	Forward: GCC*GAATTC*CGCGCCTATATCTGG	54°C	2553
	Reverse: ATGC**GTCGAC**CCCTTCGAATTGTTTC		
*Omp*	Forward: G*GAATTC*ACGCCTAAGGTTGATAT	53°C	984
	Reverse: G**GTCGAC**CTTTATCTTCTTTTGTTG		
*Apfa*	Forward: GGGC*GAATTC*ATGCAAAAACTAAGT	53°C	444
	Reverse: TATG**GTCGAC**TGATGCGCAGAAAT		

Note:

*Bam*H I site: Underlined; *Eco*R I site: Italic; *Sa*I I site: Bold; PCRs were run for 30 cycles.

The amplified fragments were cloned into pGEX-6P-1, resulting in the recombinant plasmids pGEX-apxIA, pGEX-apxIIA, pGEX-apxIIIA, pGEX-apxIVA, pGEX-apfa and pGEX-omp (for the restriction enzymes used for cloning, see Table 1). The recombinant plasmids were transformed into *E. coli *BL21 and expressed by induction with 1 mmol/L isopropyl-β-D-thiogalactoside (IPTG) under cultivation at 37°C for 4-6 h. All of the expressed recombinant proteins formed inclusions except for rOMP. The inclusion proteins were purified after denaturation and renaturation. The process involved two to three washes with 50 mmol/L Tris-HCl (pH 8.0), 1 mmol/L Ethylene Diamine Tetraacetic Acid (EDTA) containing 0.5% Triton X-100, dissolution in 6 mol/L guanidine hydrochloride, dilution, dialysis against 20 mmol/L Tris-HCl (pH 8.3), 1 mmol/L EDTA, and concentration by Polyethylene Glycol (PEG) 20 000. This was followed by redialysis against 20 mmol/L Tris-HCl (pH 8.3) and 1 mmol/L EDTA. The soluble ApxI, rApxII, rApxIII, rApxIV and rApfa as well as rOMP were purified using a MicroSpin GST Purification Module (Amersham Pharmacia Biotech Co., Piscataway, NJ, USA) according to the manufacturer's instructions. The concentration of the recombinant proteins was determined using the Bradford method as described previously [23].

### Western blotting

Western blot analysis of recombinant proteins after sodium dodecyl sulfate polyacrylamide gel electrophoresis (SDS-PAGE) was performed as described previously [23]. Rabbit antisera against APP1 (for rOMP, rApxI, rApxIV and rApfa) or APP2 (for rApxII and rApxIII) were used at a 1:50 dilution as the first antibody and horse radish peroxidase (HRP)-conjugated goat anti-rabbit Immunoglobulin G (IgG) (Sigma-Aldrich, St. Louis, MO, USA) at 1:5 000 dilution as the second antibody. 3, 3'-Diaminobenzidine (DAB) was used as the staining substrate.

### Immunization of mice

Male BALB/c mice (*n *= 80) were randomly allocated in equal numbers to each of three vaccination treatments and a PBS control, twenty mice were used in each group. The mice were immunized using 0.2 ml for each group [7] (Table 2). The immunization was performed by multipoint subcutaneous injection. The first, second and third immunizations were performed at 7, 9 and 11 weeks of age, respectively. One week after the first immunization, blood was harvested each week from the tail vein (0.1 ml/animal) for the serum antibody assay.

**Table 2:** The antigens described and vaccine components of immunized mice

Groups	vaccine components	
	antigens described	protein content
Control	total protein concentration was 100 μg/ml	rApxI, rApxII, rApxIII and rOMP
Group I	total protein concentration was 150 μg/ml	rApxI, rApxII, rApxIII, rApxIV, rOMP and rApfa
Group II	10^9 ^colony forming units (CFU)/ml	inactivated APP1 whole cell
Group III	phosphate-buffered saline (PBS)	PBS

Note:

1. APP1 inactivated with 0.3% formaldehyde solution.

2. The vaccine components of all the immunized groups as well as the control group were emulsified with an equal volume of mineral oil adjuvant (Sigma-Aldrich).

Effect of challenge with APP1 and APP2 on vaccinated mice

### Antibody analysis

Specific antibodies were measured by indirect ELISA (iELISA) [24]. Native ApxI, ApxII, ApxIII, Apfa and OMP were extracted as described previously [25-27]. Because ApxIV is expressed only in vivo during infection, it could not been extracted from the culture of APP. The crude extracts were recovered and purified by 12% SDS-PAGE. The ELISA plates (Costar, eBioscience, San Diego, CA, USA) were coated with 10 μg/ml ApxI, ApxII, ApxIII, Apfa or OMP (50 μl/well). Sera of immunized mice were diluted (1:100, 50 μl/well) with PBST (PBS with 0.1% Tween 20) as the first antibody, and HRP-conjugated goat anti-mouse IgG (Sigma Aldrich) (1:10 000 dilution, 50 μl/well) was used as the second antibody. Washing was carried out three times with PBST between each step. All reaction mixtures were set up in triplicate, and the average values were used for recording and calculation. The results were read on a Dynatech MR 7000 ELISA reader (Bio-Rad mode l680). The OD_490 _was read to record the ELISA score.

### Data analysis

The data were analyzed using the general linear model (GLM) procedure of Statistical Analysis System (SAS, 1997. Base SAS Software Reference Card. Version 6.12, Cary, NC, SAS Institute Inc., USA, p.211-253).

### Challenge after immunization

One week after the third immunization, the surviving animals in each group were subdivided again into two equal subgroups within each group. The mice in one subgroup of each group were challenged intranasally with 5 × 10^9 ^colony forming units (CFU) of APP1, and the mice in the other subgroups were challenged with APP2 (5 × 10^10 ^CFU). The LD_50 _was calculated as described previously [28]. Animals were sacrificed on the sixth day after challenge.

### Histopathology and indirect immunofluorescence (IIF) test

Lung samples were separated into two parts. One part was fixed by formalin, followed by hematoxylin and eosin (HE) staining for the observation of histological changes. Briefly, the lung of each mouse was fixed in 10% formalin, embedded in paraffin and cut into 5-6 μm sections. All sections were heated at 56°C for 25 min, deparaffinized in xylene, rehydrated with graded alcohols, and then stained with HE for histological observation using light microscopy (Olympus, Tokyo, Japan).

The other part of each lung was cut into sections using a freezing microtome for the detection of the distribution of APP in lung tissue using the IIF method [29]. Briefly, lung samples were embedded in a Tissue-Tek OCT compound (Miles, Inc., Elkhart, IN) and frozen in liquid nitrogen. Frozen sections (4 μm) were mounted on slides coated with poly-L-lysine and fixed in pre-cooled acetone for 5 min. Sections were then covered with 20 μl rabbit antiserum against APP1 or APP2 (1:50 dilution) and incubated at 37°C for 1 h. After washing in PBS, the sections were covered with 20 μl FITC-labeled goat anti-rabbit IgG (Sigma-Aldrich) (1:100 dilution) and incubated at 37°C for 1 h. Immunofluorescence images were observed with an Olympus A×70 fluorescence microscope (Olympus, Tokyo, Japan).

## Results

### Purification and concentration of the recombinant proteins

The purity of the expressed recombinant rApxI, rApxII, rApxIII, rApxIV and rApfa as well as rOMP protein was approximately 90%-95% after analysis by SDS-PAGE and thin-layer scan, and the concentrations of rApxI, rApxII, rApxIII, rApxIV, rApfa and rOMP were 150 μg/ml, 115 μg/ml, 140 μg/ml, 95 μg/ml, 80 μg/ml and 200 μg/ml, respectively.

### Detection of serum antibodies

**Figure 1:**
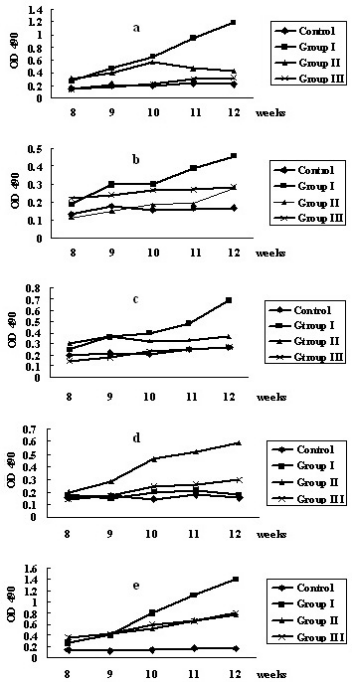
**Antibody levels against rApxI, rApxII, rApxIII, rOMP and rApfa**. 1a: ApxI, 1b:ApxII, 1c:ApxIII, 1d: Apfa, 1e: OMP

The serum antibodies to rApxI, rApxII, rApxIII, rOMP and rApfa in various groups were examined and the findings are summarized in Fig. 1.

Two weeks after the second immunization, antibodies against rApxI and rOMP in the mice in group I were significantly higher (*P *< 0.01) than those in the other three groups. Antibodies against rApxII and rApxIII were also higher in the mice in Group I than in the other three groups (*P *< 0.05). All antibody levels of the mice in Group I (against rApxI, rApxII, rApxIII and rOMP) were significantly higher (*P *< 0.01) than those in the other three groups one week after the third immunization (Table 3).

**Table 3:** 

Groups	Challenge with APP1			Challenge with APP2		
	
	Survival	Lung lesion		Survival	Lung lesion	
Control	0/10	Severe^b^		0/10	Severe^b^	
Group I	9/10	Slight^a^	Severe^b^	9/10	Slight^a^	Severe^b^
Group II	5/10	Moderate^a^	Severe^b^	6/10	Moderate^a^	Severe^b^
Group III	6/10	Moderate^a^	Severe^b^	7/10	Moderate^a^	Severe^b^

^a ^Surviving mice; ^b ^Dead mice

Antibodies against rApxI, rApxII, rApxIII and rApfa were significantly higher in the mice in Group II than in those in Group III and in the control group 2 weeks after the second immunization (*P *< 0.05). The rOMP antibody level of the mice in Group II was the same as that of Group III and these levels were significantly higher than that in the control group (*P *< 0.01). Antibody levels against rApxI, rApxII, rApxIII and rApfa in the mice in Group III were slightly higher than those in the control group but were not significantly different (*P *> 0.05).

### Mortality and histopathology

**Figure 2:**
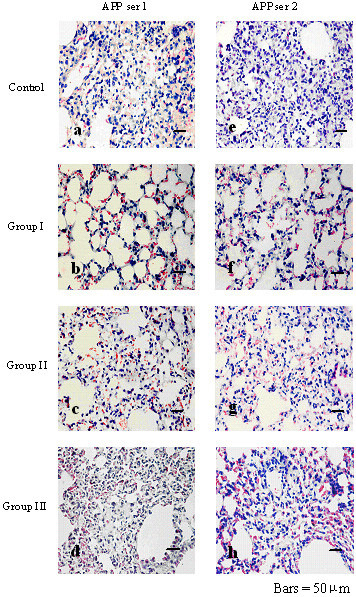
**Histopathology of lungs from mice in various groups after challenge with APP1 or APP2 (HE staining 200 × magnification)**. 2a: Control group challenged with APP1; 2b: Group I challenged with APP1; 2c: Group II challenged with APP1; 2d: Group III challenged with APP1; 2e: Control group challenged with APP2; 2f: Group I challenged with APP 2; 2g: Group II challenged with APP2; 2h: Group III challenged with APP2.

The challenge doses of APP1 Shope 4074 and APP2 S1536 were 5×10^9 ^cfu and 5×10^10 ^cfu respectively. Within 24 h after challenge with APP1 and 36 h after challenge with APP2, all control mice died. The survival rate of Group I was higher than that of Groups II and III (*P <*0.05) and the control group (*P <*0.01). The results are summarized in Table 2. Bleeding from the mouth and nose was apparent in all dead mice. The lungs of the dead mice challenged with APP1 (Fig. 2a) and APP2 (Fig. 2e) showed severe lung lesions. Congestion, hemorrhage, necrosis and parenchyma consolidation were observed in the lungs, and extensive serous and fibrinous exudates had accumulated together with a substantial infiltration of inflammatory cells. All the other surviving mice were euthanized 5 days post challenge with APP1 or APP2. The mice in Group I challenged with APP1 (Fig. 2b) or APP2 (Fig. 2f) had less severe lung lesions than those in Groups II and III (Fig. 2c, 2d, 2g, 2h), with less hemorrhage and necrosis. The mice in Groups II and III showed moderate lung lesions, with pulmonary congestion, hemorrhage, serous and fibrinous exudation in some areas, inflammatory cell infiltration, as well as partial rupture of alveolar structures, and lysis.

### IIF detection

The results of IIF detection are shown in Table 4. In Group I, there were only weak and scattered fluorescent dots observed in individual alveoli and alveolar septa of the surviving mice. In contrast, in those in groups and Group III, the fluorescence dots were more dense and stronger than in Group I. However, the strongest fluorescence was observed in most alveoli and alveolar septa of the dead mice in the control group as well as those in Groups I-III.

**Table 4:** Detection of APP1 or APP2 in the lungs of mice by indirect immunofluorescence (IIF)

Groups	Detection of APP			
	Challenge with APP1		Challenge with APP2	
Control	++^b^		++^b^	
Group I	±^a^	++^b^	±^a^	++^b^
Group II	+^a^	++^b^	+^a^	++^b^
Group III	+^a^	++^b^	+^a^	++^b^

Fluorescence intensity: Weak: ±; Medium: +; Strong: ++ [29]

^a ^Surviving mice; ^b ^Dead mice

## Discussion

This study showed that a recombinant subunit vaccine consisting of rApxI, rApxII, rApxIII and rOMP can protect mice effectively against challenge with APP1 and APP2. This demonstrates that the recombinant subunit vaccine can induce favorable cross-protection. Compared with this, the cross-protection efficacy of the inactivated vaccine (Group III) was significantly lower than that in Group I. This may be due to the lower antibody level against Apx toxin in group III, which indicates the importance of Apx toxin for cross-protection [2,3,6]. The results showed that the antibodies against rApxI, rApxII, and rApxIII in Group I were higher than those in the other groups, which could have contributed to the better protection of this group. Furthermore, with increasing in the time since immunization, the antibody levels also increased, especially the antibodies against rApxI and rOMP; there was a large rise between the second and third immunization. Because ApxIV toxin has been shown to be produced only in vivo, it can not be extracted from cultures to design diagnostic tools from a culture of APP to use as the diagnostic antigen in iELISA. Therefore, we did not detect the antibody titer of rApxIV. However, the positive effect of rApxIV on the immune response is reflected in the results of the challenge experiment [30].

During these experiments, we showed that the protective efficacy of the vaccine did not improve against APP1 and APP2 after rApfa was added to the vaccine containing rApxI, rApxII, rApxIII and rOMP. Instead the protective efficacy was decreased, suggesting that the protective efficacy was lower than before. Suggesting that rApfa may just have a negative effect when combined with other factors in Group II, the antibody titers against rApxI, rApxII, rApxIII and rOMP decreased following the addition of rApfa. It is interesting that, the antibody titers against rApxI and rApxIII declined with an increase in the time since immunization. We propose that the rApfa may impair immunity or rApfa antibody counteracted the other antibodies to rApxI, rApxII, rApxIII and rOMP in Group II. However, we determined the antibody titer of rApfa, and the result showed that it rose slowly along with the increase in time since immunization. These results are similar to those of a previous study, in which the protective efficacy of a subunit vaccine containing three antigens (PalA, ApxI and ApxII) was considerably lower than that containing two antigens (ApxI and ApxII). This could indicate that PalA antibody counteracted ApxI and ApxII antibodies, and thus interfered with immunity [9].

In summary, rApfa interfere with the other antibodies against toxins of APP. Consequently, the fluorescence dots in group II were more dense and stronger than in group I and the mice in group II challenged with APP1 or APP2 had more severe lung lesions than those in group I. In addition, the survival rate of Group II was lower than that of Group I. It indicated that there was no positive correlation between the quantity of multicomponent recombinant vaccines antigen components and immune protection, the optimization of the antigen components was the key to a better immune protection.

Finally, which component of rApfa may interfere with immunity when mixed with other antigens should be studied further. After all, the mouse is only a model for this study.

## Conclusion

The result of this study indicates that the multicomponent recombinant subunit vaccine composed of rApxI, rApxII, rApxIII and rOMP can provide effective cross-protection against challenge with APP1 and APP2.

## Competing interests

The authors declare that they have no competing interests.

## Authors' contributions

MLS, YW and CLW carried out the study. YG and YGP carried out the molecular genetic studies. JDL participated in the sequence alignment. MLS, CLW and SGL drafted the manuscript. MLS and YW carried out the immunoassays and the clinical examinations. GXL and HFL performed the statistical analysis. SGL designed the study. All authors read and approved the final manuscript.
